# Psychological, social, and mechanical work exposures and disability retirement: a prospective registry study

**DOI:** 10.1186/s12889-016-3921-0

**Published:** 2017-01-10

**Authors:** Jan S. Emberland, Morten Birkeland Nielsen, Stein Knardahl

**Affiliations:** Department of Work Psychology and -Physiology, National Institute of Occupational Health, P.O. Box 8149 Dep, N-0033 Oslo, Norway

**Keywords:** Disability retirement, Occupational, Psychosocial, Mechanical, Registry data, Prospective, Hazard ratio

## Abstract

**Background:**

Relations between several occupational psychological and social factors and disability retirement remain largely unexplored. Knowledge of which specific aspects of the work environment that affect risk of disability is a prerequisite for the success of organizational interventions aiming to prevent premature work force exit. The objective of the present study was to determine contributions to registered disability retirement by a broad range of psychological and social work exposures while taking into account effects of mechanical exposure.

**Methods:**

Written consent was obtained from 13 012 employees (96 organizations) representing a wide range of occupations, to link their survey responses to data from the Norwegian national registry of disability compensation. Median follow-up time was 5.8 years. To determine effects of self-reported work exposures on risk of disability retirement hazard ratios (HR) and 99% confidence intervals (99% CI) were calculated with Cox regression analysis. Effects of sex, age group, skill level, sickness absence in the last three years, and work exposures estimated to be confounders were accounted for. Post hoc stratification by sex was conducted to explore if identified predictors affected risk of disability retirement differently in men compared to women.

**Results:**

Contributors to higher risk of disability retirement were “role conflict” (high level HR 1.55 99% CI 1.07 to 2.24) and “physical workload” (high level HR 1.93 99% CI 1.39 to 2.68). Contributors to lower risk of disability retirement were “positive challenge” (high level HR 0.56 99% CI 0.34 to 0.93), “fair leadership” (high level HR 0.56 99% CI 0.39 to 0.81), and “control over work intensity” (high level HR 0.62, 99% CI 0.47 to 0.82). Direction of effects was not dependent on sex in any of the five identified predictors.

**Conclusions:**

Several specific psychological and social work factors are independent contributors to risk of disability retirement. In order to prevent premature work force exit workplace interventions should consider targeting the predictors identified by the present study.

## Background

Exit from working life due to disability incurs large costs for societies as well as challenges to the quality of life of persons. Premature exit may result from impacts of biological/medical, psychological, and social conditions on functioning [1]. Still, the potential influences of a variety of non-physical work environment conditions on disability retirement have previously been devoted little attention. The present study aimed to determine which of a broad range of specific occupational psychological and social factors that may contribute to disability retirement.

The workplace is an arena where individuals face challenges inherent in work tasks and social interactions. Work also provides opportunities for positive experiences from achievement and friendship. For many people, the job is a major source of feedback, fulfillment, and personal identity which in turn may promote work motivation, health, and work ability. Hence, conditions at work may contribute to disability retirement in several ways, e.g. by (I) influencing health and work ability, by (II) influencing motivation to work, or by (III) making work too demanding relative to work ability (competence) in the jobs that are available.

A 2011 systematic review of psychological and social work factors contributing to disability retirement [2] revealed that only a limited number of factors have been examined. The majority of the reviewed studies sought to identify contributions of “low control,” “psychological demands,” and “social support.” In many of these, as well as subsequent studies on registered work disability (e.g. [3–5]) it is apparent that the Job Content Questionnaire (JCQ; [6]) has been the preferred instrument to measure psychological and social work factors. This instrument groups several specific factors under broad demand and control dimensions, which raises the possibility that factors with opposite effects are grouped together under the same heading. However, knowledge of specific contributors to disability is arguably a prerequisite for developing practical interventions at the workplace.

Recent studies have shown that psychological and social factors other than the dimensions assessed with the JCQ instrument contribute to health symptoms (e.g. [7, 8]) and perceived work ability [9]. Hence, it seems timely to broaden the scope by exploring contributions to disability retirement by a wider range of non-physical work factors.

Biomechanical workload has received considerable attention in relation to registered work disability, and several studies have examined effects of both mechanical and psychological/social work factors [4, 5, 10, 11]. Based on previous publications it remains somewhat unclear if mechanical workload plays an important role in explaining associations between non-physical occupational factors and work disability. While the main objective was to determine contributions of a broad range of psychological and social work factors to publicly registered disability retirement, the present study also sought to account for effects of mechanical work exposure.

## Methods

### Study design

The current study is part of a full-panel prospective study of work factors contributing to health, work ability, absence, and exit from working life in Norway. Employees were invited to participate in a web-based survey containing questions on background information, psychological, social, and mechanical work factors, work organization, mastery of work, attitudes towards work, organizational change, personality, health behavior, coping strategies, mental health, health complaints, and work ability. Each employee received a letter containing information about the survey and a personalized code for logging into the web-based questionnaire. A paper version of the questionnaire was made available upon request. Written information specified the strict confidentiality guidelines and informed about the license for data collection granted by the Norwegian Data Inspectorate. The organizations from which employees were recruited provided data on employees’ departmental affiliation, home address, and occupational title according to the Norwegian standard classification of the occupations (STYRK) - a system developed by Statistics Norway based on the International Classification of Occupation (ISCO-88). In return for participation in the project, the organizations received written reports and oral presentations of results with the objective of supporting management and personnel in the process of monitoring their work conditions.

### Population

**Fig. 1:**
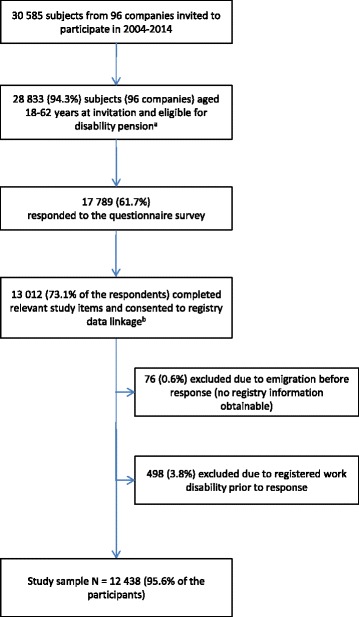
Flow diagram depicting the selection process. ^a^Employees above 62 years of age are additionally eligible for statutory early age pension and thus excluded from the present study. ^b^National registers maintained by the Norwegian Labour and Welfare Administration

Employees were recruited from 96 companies representing a broad spectrum of occupational sectors including health care, education, government and public administration, engineering, project management, industry, and non-profit organizations. A total of 30 585 subjects were invited to participate in the period 2004 to 2014. At the time of invitation 28 833 (94.3%) subjects were aged 18–62 years and thus eligible for disability retirement benefits. Subjects above the age of 62 were excluded as they are additionally entitled to early age pension. Of the invited aged 18-62, 17 789 (61.7%) responded to the questionnaire survey. Written consent was obtained from 13 012 (73.1%) subjects and enabled linking these individuals’ questionnaire responses to data from the national registry of disability compensation maintained by the Norwegian Labour and Welfare Administration (NAV). Based on information from this registry subjects were excluded from the present study if having incomplete history of work disability (emigration before response, *n* = 76; 0.6%) or having received disability retirement compensation (due to some proportion of disability) prior to response (*n* = 498; 3.8%). Thus, the final cohort (Fig. 1) consisted of 12 438 subjects (95.6% of the participants; mean age 41.8 years; 55.5% women) with a median follow-up time of 5.8 years.

### Ethical approval

The project has been approved by the Regional Committees for Medical and Health Research Ethics (REC) in Norway and has permission from The Norwegian Data Protection Authority. The research was carried out in compliance with the World Medical Association Declaration of Helsinki and written informed consent was obtained from all study participants (for details see [12]).

### Outcome: disability retirement

Disability retirement was defined as the subject receiving disability pension compensation from the Norwegian Labour and Welfare Administration (NAV). Unlike reimbursement for sickness absence, the requirement for receiving disability pension is that the magnitude of work inability is at least 50% [13]. Compensation criteria must be substantiated by an extensive physician’s certificate and confirmed by examinations (which includes assessment of job specific capacities) undertaken by specialized (usually medical) representatives from a local NAV office. For the respondents consenting to registry linkage we had access to information on disability pension compensation recorded in the NAV registry up to 1 January 2015 (the end of the present study’s follow-up period).

### Psychological, social, and mechanical work factors

Psychological and social exposures were measured by the General Nordic Questionnaire for psychological and social factors at work (QPS_Nordic_). Psychometric evaluations of QPS_Nordic_ have shown high validity and reliability of the scales included in the present study [14]. Reliability analyses indicate that the factors are consistent across a wide range of occupational groups [15].

The psychological and social scales covered job demands, control at work, predictability at work, role expectation, leadership, and organizational culture and climate (see Table 3). A complete list of scale items has been published elsewhere [14, 15]. Responses to items were given on a 5-point scale; 1 “very seldom or never,” 2 “somewhat seldom,” 3 “sometimes,” 4 “somewhat often,” and 5 “very often or always.”

Mechanical exposure was measured with two items; *physical workload*, and *working with arms raised to or above shoulder level* (single item). *Physical workload* was assessed by measuring the extent to which subjects were lifting or handling objects that weigh approximately 1–5 kg, 6–15 kg, and more than approximately 15 kg with own muscular strength. Response categories for both measures were; 1 “seldom or never,” 2 “sometimes,” 3 “daily,” and 4 “many times per day.”

To address dose-response relationships, scale scores were categorized into three exposure levels. In line with previously published analyses from parts of the same cohort [8, 9] scores from 1.00 through 2.60 were classified as “low”; 2.61 through 3.40 as “middle”; and 3.41 through 5.00 were classified as “high” exposure, respectively. For the four-level measures (mechanical exposures) scores from 1.00 through 2.50 were classified as “low”; 2.51 through 3.50 as “middle,” and 3.51 through 4.00 as “high” exposure, respectively.

### Statistical analysis

Statistical analyses were conducted with SPSS version 22.0 (IBM, Armonk, NY) and with the survival package [16] for R version 3.2.2.

Non-response analysis was conducted to determine if background variables influenced whether subjects responded at baseline. Binary logistic regressions were run with response status as outcome. The effects of age, sex, occupational group, and skill level were estimated separately. Since the occupational group variable does not have a clear intrinsic order this variable was treated as nominal. Thus, the effects of each of the 10 occupational categories on response status were calculated by using the combination of all other occupational groups as reference.

Hazard ratios were calculated with Cox regression analysis to determine effects of background variables and 16 work exposures on disability retirement. Analyses were run separately with each background/work exposure variable as independents. Since multiple analyses were performed 99% confidence intervals were chosen in order to reduce the risk of type 1 error. Due to the clearly higher hazard ratio for subsequent work disability in women (Table 2) post hoc analysis with stratification by sex was conducted to uncover if the identified predictors in the primary analysis (see ﻿Table 3)﻿ were differentially related to work disability according to sex. In order to calculate a single effect estimate for each predictor (to be compared across sexes), exposure variables were entered as continuous in these analyses.

As recommended for studies of healthy populations [17] attained age (at censoring/event) was the underlying time scale in the analyses (except in the non-response analysis with age group as the primary predictor) rather than “time-on-study” (i.e. years since baseline response).

Interactions between each background/work exposure variable and the logarithm of the follow-up time i.e. age in years, (for age group; time since response) were determined by tests of non-zero slopes followed by graphical plots of scaled Schoenfeld residuals. The tests indicated no violations of the proportional hazards assumption (for all variables: *P* >0.05).

Follow-up time was calculated from the calendar date subjects responded to the web-based questionnaire. For subjects completing the paper version of the questionnaire (*n* = 1106) and for some of the web responders (*n* = 882) information on actual response date was not obtainable. In these cases response date was set to the last possible date of response for all employees in their respective company. Follow-up ended at the time point of being granted disability pension (*n* = 553), emigration (*n* = 42), death (*n* = 39), or 1 January 2015 (*n* = 10 407) whichever came first. At the time of this study data from the old age pension registry had not been obtained. Thus, subjects were also censored if they reached the eligible age for early statutory pension (which is the first day of the month following the 62nd birthday) before the end of follow-up (*n* = 1397).

#### Potential confounding variables

The effects of sex, skill level, age group, and sickness absence (in the 3 years preceding baseline response) were accounted for in all analyses of work exposures and disability retirement. Skill level was determined by recoding the occupational groups (ISCO-88) in agreement with the International Standard for Classification of Education (ISCED). In cases where no information on occupational group (ISCO-88) had been provided by the subjects’ respective companies, missing values were substituted with self-reported skill level information (*n* = 102). Baseline age was categorized into; 18–29, 30–39, 40–49, and 50–62 years, to account for potential cohort effects [17].

Some chronic illnesses may lead to reductions in certain work exposure levels [18, 19]. Also, a number of chronic diseases are linked with risk of disability pensioning [20]. Thus, the analyses were adjusted for sickness absence prior to baseline response caused by cardiovascular disease, respiratory illnesses, cerebrovascular disease, diabetes, or cancer [21, 22]. This information was obtained from the sickness absence registry maintained by the Norwegian Labour and Welfare Administration and limited to the 3 years prior to baseline participation.

The observed effect of each work exposure on work disability may be confounded by the effects of other work exposures. However, since the scales are interrelated [14, 15] simultaneously controlling for all other work exposures is likely to result in overadjustment. Thus, a strategy described by Rothman et al. [23] was utilized to identify influence by other work exposures above a certain threshold level. First, the effect of an exposure on disability retirement was estimated. Subsequently, one other exposure was added to the model. If the effect estimate of the original exposure changed by more than 10% in the second model the added exposure was defined as a confounder [23]. This procedure was carried out for each of the 16 work exposure measures. The influence of *each* of the other 15 work exposure measures was estimated.

## Results

### Non-response analysis

Sex was not predictive of responding at baseline (*P* >0.05; Table 1). Subjects aged >29 (i.e. 30–39, 40–49, and 50–62) exhibited higher odds of responding compared to the lowest age group (for all: *P* <0.05). The non-response analyses further revealed that the groups; *legislators/senior officials/managers*, *professionals*, and *clerks* exhibited significantly *higher* odds of responding at baseline compared to all other occupational classes (combined). Lower odds of responding were detected for *service workers/shop/market sales workers* and *plant/machine operators/assemblers*. The analyses of skill level indicated that subjects with competence level corresponding to less than 4 years of higher education exhibited lower likelihood of responding at baseline. Subjects with unspecified competence level (this being legislators/senior officials/managers) had a clearly higher likelihood of responding compared to subjects with competence level equivalent to minimum 4 years of higher education.

**Table 1:** Sample characteristics and their associations with baseline response

	Employees aged 18–62 years (*N* = 28 833)				Association with baseline response		
Background variable	Participants (*n* = 13 012)		Non-participants (*n* = 15 821)				
	N	%	N	%	OR	95% CI	*P*-value
Sex							
Male	5733	44.06	7108	44.93	1.00		
Female	7279	55.94	8669	54.79	1.04	0.99–1.09	0.091
Missing data	0	0	44	0.28			
Age in years, mean (SD)	41.91 (10.20)		40.43 (11.19)				
<30	1725	13.26	3228	20.40	1.00		
30–39	3791	29.13	4397	27.79	1.61	1.50–1.74	<0.001*
40–49	4078	31.34	4222	26.69	1.81	1.68–1.94	<0.001*
50–62	3418	26.27	3974	25.12	1.61	1.49–1.73	<0.001*
Classification of occupation^a^							
Legislators, senior officials, and managers	1120	8.61	580	3.67	1.83	1.65–2.03	<0.001*
Professionals	3662	28.14	2837	17.93	1.25	1.18–1.32	<0.001*
Technicians and associate professionals	3641	27.98	3336	21.09	0.99	0.94–1.05	0.797
Clerks	728	5.59	527	3.33	1.28	1.14–1.43	<0.001*
Service workers and shop and market sales workers	2940	22.59	3626	22.92	0.66	0.63–0.70	<0.001*
Skilled agricultural and fishery workers	1	< 0.01	7	0.04	0.13	0.02–1.06	0.056
Craft and related trades workers	394	3.03	394	2.49	0.91	0.79–1.05	0.186
Plant and machine operators and assemblers	44	0.34	95	0.60	0.42	0.29–0.60	<0.001*
Elementary occupations	322	2.47	312	1.97	0.94	0.81–1.10	0.435
Armed forces and unspecified	0	0	1	<0.01	-		-
Missing data	160	1.23	4106	25.95			
Skill level^b^							
Competence equivalent to minimum 4 years of higher education (>15 years)	3675	28.24	2848	18.00	1.00		
Competence equivalent to 1–3 years of higher education (13–15 years)	3683	28.30	3373	21.32	0.85	0.79–0.91	<0.001*
Competence equivalent to high school (10–12 years)	4150	31.89	4713	29.79	0.68	0.64–0.73	<0.001*
Occupations that do not require high school (<10 years)	336	2.58	324	2.05	0.80	0.69–0.94	0.008*
Occupations with unspecified requirements for competence	1120	8.61	581	3.67	1.49	1.34–1.67	<0.001*
Missing data	48	0.37	3982	25.17			

*N* number of subjects, *OR* odds ratio, *CI* confidence interval, *SD* standard deviation

^a^Treated as binary variables. Each occupational group coded as 1 while all other listed occupational groups coded as 0 (reference) in each analysis

^b^Occupations grouped into the level of competence expected for the respective occupations. In cases with no information from company records missing values were substituted by, if available, self-reported skill level

* *P* < 0.05

### Background characteristics predictive of disability retirement

Women exhibited a higher risk of disability retirement compared to men (*P* <0.05; Table 2). Being in the age group 50 to 62 was associated with an increase in risk of disability retirement compared to being <30 years of age. Also, differences in risk of disability retirement were found between the occupational groups; *service workers/shop/market sales workers* exhibited *higher* risk compared to the other occupational groups combined*. Legislators/senior officials/managers*, *professionals,* and *craft and related trades workers* showed *lower* risk. In the analyses of skill level, lower levels were associated with higher risk of disability retirement. One exception was the group with unspecified requirements for competence level (this being legislators/senior officials/managers) which showed a lower risk of disability retirement compared to the reference group (subjects with minimum 4 years of higher education). Finally, subjects with sickness absence due to a range of chronic diseases registered in the 3-year period prior to baseline response exhibited higher risk of disability retirement (*P* <0.05).

**Table 2:** Baseline sample characteristics as predictors of disability retirement

Background variable	Number	Disability cases n	%^a^	HR^b^	95% CI	*P*-value
Sex						
Male	5536	111	2.01	1.00		
Female	6902	442	6.40	3.30	2.68–4.06	<0.001*
Age in years, mean (SD)	41.79 (10.23)	44.02 (9.80)				
<30	1697	58	3.42	1.00		
30–39	3641	117	3.21	0.88	0.64–1.20	0.416
40–49	3863	184	4.76	1.34	0.99–1.80	0.051
50–62	3237	194	5.99	2.31	1.72–3.09	<0.001*
Classification of occupation^c^						
Legislators, senior officials, and managers	1101	26	2.36	0.35	0.23–0.51	<0.001*
Professionals	3510	112	3.19	0.62	0.51–0.77	<0.001*
Technicians and associate professionals	3476	153	4.40	1.04	0.86–1.25	0.700
Clerks	690	46	6.67	1.30	0.96–1.76	0.092
Service workers and shop and market sales workers	2794	173	6.19	2.26	1.89–2.71	<0.001*
Skilled agricultural and fishery workers	1	0	0	-	-	-
Craft and related trades workers	358	4	1.12	0.26	0.10–0.70	0.008*
Plant and machine operators and assemblers	39	3	7.69	1.72	0.55–5.36	0.348
Elementary occupations	316	22	6.96	1.52	0.99–2.32	0.056
Armed forces and unspecified	0	0	0	-	-	-
Missing data	153	14	9.15			
Skill level^d^						
Competence equivalent to minimum 4 years of higher education (>15 years)	3523	113	3.21	1.00		
Competence equivalent to 1–3 years of higher education (13–15 years)	3517	156	4.44	1.48	1.16–1.88	0.002*
Competence equivalent to high school (10–12 years)	3924	232	5.91	2.23	1.78–2.79	<0.001*
Occupations that do not require high school (<10 years)	329	24	7.29	2.16	1.39–3.35	0.001*
Occupations with unspecified requirements for competence	1101	26	2.36	0.53	0.35–0.82	0.004*
Missing data	44	2	4.55			
Sickness absence^e^						
No	11911	493	4.14	1.00		
Yes	527	60	11.39	2.17	1.66–2.83	<0.001*

*N* number of subjects, *HR* hazard ratio, *CI* confidence interval, *SD* standard deviation

^a^Percentage of registered disability pension cases within each category

^b^Crude effect

^c^Treated as binary variables. Each occupational group coded as 1 while all other listed occupational groups coded as 0 (reference) in each analysis

^d^Occupations grouped into the level of competence expected for the respective occupations. In cases with no information from company records missing values were substituted by, if available, self-reported skill level

^e^Due to respiratory illnesses, cardiovascular disease, cerebrovascular disease, diabetes, or cancer in the 3 years prior to response

* *P* < 0.05

### Work exposures predictive of disability retirement

Higher risk of disability retirement was associated with the highest baseline levels of *role conflict* and *physical workload* (*P* <0.01; Table 3). Lower risk of disability retirement was predicted by the highest baseline levels of *positive challenge*, *control over work intensity*, and middle and high level of *fair leadership* (*P* <0.01).

**Table 3:** Psychological, social, and mechanical work factors as predictors of disability retirement

Factor	Number	HR^a^	99% CI	*P*-value for trend
Decision demands				0.411
Low	986	1.00		
Middle	4890	0.82	0.56–1.21	
High	5958	0.86	0.58–1.26	
Quantitative demands				0.035
Low	4012	1.00		
Middle	4686	1.14	0.87–1.49	
High	3318	1.28	0.95–1.73	
Role clarity				0.913
Low	386	1.00		
Middle	1478	0.60	0.30–1.18	
High	10283	0.70	0.39–1.25	
Role conflict				0.020
Low	5797	1.00		
Middle	5042	1.25	0.99–1.59	
High	1312	1.55	1.07–2.24	
Positive challenge^b^				0.027
Low	440	1.00		
Middle	1983	0.68	0.40–1.17	
High	8675	0.56	0.34–0.93	
Control over work intensity				<0.001
Low	3461	1.00		
Middle	2406	0.84	0.62–1.14	
High	6227	0.62	0.47–0.82	
Decision control^c^				0.005
Low	4057	1.00		
Middle	4374	0.92	0.70–1.20	
High	3135	0.70	0.48–1.01	
Predictability during the next month				0.998
Low	568	1.00		
Middle	1423	1.70	0.88–3.32	
High	10159	1.22	0.66–2.23	
Support from immediate superior^d^				0.826
Low	989	1.00		
Middle	2517	1.06	0.67–1.67	
High	8343	0.96	0.60–1.52	
Empowering leadership				0.175
Low	2930	1.00		
Middle	4260	0.80	0.60–1.05	
High	4935	0.86	0.65–1.13	
Fair leadership				0.001
Low	753	1.00		
Middle	2175	0.61	0.40–0.93	
High	9064	0.56	0.39–0.81	
Innovative climate^bde^				0.035
Low	787	1.00		
Middle	3703	0.85	0.53–1.37	
High	6484	1.04	0.64–1.71	
Social climate^de^				0.528
Low	584	1.00		
Middle	2947	0.89	0.53–1.50	
High	7826	0.92	0.54–1.56	
Human resource primacy				0.110
Low	2886	1.00		
Middle	4566	0.79	0.59–1.04	
High	4210	0.79	0.59–1.05	
Physical workload^c^				<0.001
Low	7115	1.00		
Middle	3075	1.26	0.94–1.68	
High	1631	1.93	1.39–2.68	
Working with arms raised to or above shoulder level^cf^				0.058
Low	8040	1.00		
Middle	2434	1.15	0.84–1.58	
High	1314	1.21	0.83–1.76	

*N* number of subjects, *HR* hazard ratio, *CI* confidence interval

^a^Adjusted for sex, age group, skill level, and sickness absence in the 3 years prior to response caused by cardiovascular disease, respiratory illnesses, cerebrovascular disease, diabetes, or cancer

^b^Adjusted for decision control

^c^Adjusted for control over work intensity

^d^Adjusted for fair leadership

^e^Adjusted for human resource primacy

^f^Adjusted for physical workload

Post hoc stratification by sex with the five identified contributors revealed that the risk estimates for women and men were in the same direction for all of the predictors (Table 4). Also, differences in the hazard risk ratios were minor comparing the sex-specific effects of *role conflict* and *control over work intensity*. With a risk ratio difference of more than 10% two of the factors were notably more protective of disability retirement in men i.e. *positive challenge* (HR_men_ 0.77, CI 0.54–1.11 vs HR_women_ 0.96, CI 0.80–1.15) and *fair leadership* (HR_men_ 0.74, CI 0.57–0.96 vs HR_women_ 0.86, CI 0.75–0.99). Further, with a risk ratio difference of 26.2% *physical workload* was a stronger risk factor to disability retirement in men than in women (HR_men_ 1.68 CI 1.22–2.32 vs HR_women_ 1.24 CI 1.06–1.45). Although differences in magnitude of effects were detected the confidence intervals for all factors overlapped in range across the sexes.

**Table 4:** Psychological, social, and mechanical factors as predictors of disability retirement in men and women

	Men				Women				
Factor^a^	N	HR^bc^	99% CI	*P*-value	N	HR^bc^	99% CI	*P*-value	% difference^d^
Role conflict	5426	1.33	0.98–1.82	0.017	6725	1.26	1.08–1.48	<0.001	5.26
Positive challenge^e^	5026	0.77	0.54-1.11	0.065	6072	0.96	0.80–1.15	0.527	19.79
Control over work intensity	5398	0.83	0.64–1.09	0.081	6696	0.81	0.72–0.92	<0.001	2.41
Fair leadership	5354	0.74	0.57–0.96	0.003	6638	0.86	0.75–0.99	0.005	13.95
Physical workload^f^	5288	1.68	1.22–2.32	<0.001	6533	1.24	1.06–1.45	<0.001	26.19

*N* number of subjects, *HR* hazard ratio, *CI* confidence interval

^a^Scale scores ranging from 1 to 5. For physical workload; 1 to 4

^b^Risk of disability retirement per unit increase in each of the listed work exposures. HRs and CIs calculated separately for men and women

^c^Adjusted for age group, skill level, and sickness absence last 3 years prior to baseline response caused by cardiovascular disease, respiratory illnesses, cerebrovascular disease, diabetes, or cancer

^d^Percentage difference in HRs between men and women

^e^Adjusted for decision control

^f^Adjusted for control over work intensity

## Discussion

The present results clearly indicated that in addition to physical workload higher levels of several psychological and social work exposures independently predict subsequent disability retirement. A *lower* risk of disability retirement was predicted by the factors *positive challenge*, *control over work intensity*, and *fair leadership. Role conflict* and *physical workload* were the most prominent predictors of *higher* risk of disability retirement. Of these five factors *positive challenge, fair leadership,* and *physical workload* seemed to be more strongly related to disability retirement in men compared to women. In magnitude of effects, *role conflict* and *control over work intensity* revealed to be somewhat equally important to disability retirement across the sexes.

Previous studies investigating relations between psychological/social work exposures and disability retirement have often employed the “demand-control” framework. When outlining the job demand and job control dimensions, Karasek [24] stated that “a correct analysis must distinguish between two important elements of the work environment at the individual level: (1) the job demands placed on the worker and (2) the discretion permitted the worker in deciding how to meet these demands” (p. 285). Hence, the demand and control dimensions incorporate a wide range of work environment aspects. Measured with the JCQ, [6] “job demands” and “decision latitude” have repeatedly been examined to elucidate potential separate effects of these dimensions on risk of disability retirement. Across studies of occupationally diverse populations, however, consistency of findings seems low [3–5, 25, 26].

Results from the present study may provide some explanation to the previous inconsistencies. *Decision demands*, q*uantitative demands*, and *role conflict* reflect aspects which are grouped together in the JCQ’s job demands-dimension. While *role conflict* was identified as a risk factor to disability retirement, the effects of *decision demands* and q*uantitative demands* revealed to be of less influence. To some extent, opposing effects were also found. Although not significantly, higher *decision demands* tended to be *protective* of disability retirement. Hence, complex decision-making and high attention demands seem to impact risk of disability retirement very differently from frequent experiences of role conflicts (e.g. incompatible requests from two or more people).

“Skill discretion” is one of two components covering JCQ’s “decision latitude” [6] but few previous studies have addressed the potential independent effect of this aspect on disability retirement (see [27] for an exception). This is somewhat surprising since use of competence almost seems inseparable from the concept of “ability to perform work” [28]. *Positive challenge* (e.g. skills/knowledge useful in work) was identified as a clearly protective factor in the present study, a finding that arguably adds to the current state of knowledge on precursors to disability retirement.

The current study tested predictions of the leadership factors *fair*, *empowering*, and *supportive leadership*. Recently, an index comprising elements from *fair leadership* (i.e. “immediate superior treat employees fair and impartially”) and *supportive leadership* (e.g. “get support/help from immediate superior”) was found consistently predictive of (self-reported) disability retirement in a randomly drawn Norwegian cohort [29]. Investigating the *independent* effects of the three above-mentioned leadership factors only *fair leadership* was identified as a substantial protective factor by the present study. The indicated non-significant effect of *supportive leadership* seems, however, to resonate with some earlier findings [30, 31].

There seems to be a paucity of studies investigating the potential discriminating effects of different sources of support on disability retirement. On the contrary, joint effects of leadership and co-worker support have repeatedly been reported and found statistically non-significant [3–5]. In the present study, the *social climate* factor encompasses support from within the work unit (e.g. “encouraging and supportive work unit climate”). However, none of the factors encompassing support at work (*support from immediate supervisor* and *social climate*) exhibited statistically significant effects on risk of disability retirement. Still, differential impact of sources of support remains an important issue as the social climate measure was designed to address aspects other than co-worker support *per se*.

The distributions of some of the work exposures were fairly skewed in a negative direction. In these exposures, three-level categorization resulted in relatively few observations in the reference category. This may have inflated confidence intervals. Issues with categorization of *role clarity* have previously been noted [15] and trichotomizing of this exposure may not have been optimal to detect statistically significant effects on disability retirement. This issue may also concern the observed negative but non-significant effect of *predictability during the next month*. It seems unclear, however why higher levels of predictability would contribute to an increased risk of subsequent disability retirement. One plausible explanation may be that subjects with non-optimal work ability-level, and on the path towards disability retirement, hold jobs in which working conditions are fairly stable across time. Alternatively, job tasks and the general organization of work may have been made more predictable for subjects at risk of retiring due to health problems. Thus, the presence of “reverse” effects cannot be ruled out. However, the present analyses were adjusted for incidences of previous sickness absence due to chronic diseases in order to make “reverse” effects a less of a concern when interpreting the results.

Previous investigations do not seem to reveal substantial sex differences in the effects of psychological and social factors on subsequent disability retirement [3, 32]. The present results suggested that some of the specific work exposures may affect the disability risk of men stronger than the risk of women. In particular, the effects of physical workload seemed considerably stronger in men. One might suspect unequal distribution (across the sexes) of physical job demands (see e.g. [33]) to be accountable for such a finding. However, the rate of men (14.7%) and women (13.0%) reporting “high” physical workload were fairly equal in the current sample (analysis not shown). Also, analyses were adjusted for “skill level” which should have helped partialling out effects of occupational differences. Although some differences in magnitude of effects were discovered, all effect estimates were in the same direction across the sexes. Thus, the present findings do not provide any particular support for the notion of differentiating on sex when selecting work factors to be targeted in work place interventions.

### Methodological considerations

The participants were recruited through recruiting of businesses and possibly, not all employees were sufficiently encouraged and/or reminded to participate. A higher response rate, however, would not automatically enhance the *external* validity (i.e. generalizability) of the present results. The diverse sample covered a wide range sectors and occupations. Still, only a randomly drawn sample from the entire working population would allow generalization of the results to the (Norwegian) working population at large.

The response rate may have compromised the *internal* validity of the study if, for instance, highly exposed subjects - independent of their subsequent disability status - were less likely to participate, *and*, subjects ending up with disability pension also tended to avoid participation at baseline (due to e.g. health issues) [34]. The disability risk rate of women compared to men was in concordance with that reported in a representative Norwegian sample [29] and the numbers of new disability cases per year were comparable. Thus, it is not suspected that participants which during follow-up received disability pension were over or underrepresented in the present population. Also, previous investigations in comparable studies suggest participation not to be substantially affected by health status [35, 36].

According to the non-response analysis the background characteristics age, occupational class, and skill level seem to influence baseline participation status. It is unclear however if non-response based on these variables may have posed a threat to the validity of the current findings. This could be the case if these characteristics influenced the observed exposure–outcome associations. Accounting for the effects of background characteristics in the main analyses should, however, have helped control the potential influence of (self) selection bias [37].

The fact that exposure data (self-report) and outcome data (the national registry of disability compensation) were gathered by different methods allows for ruling out many of the sources of common method bias [38] as likely determinants of the observed associations. Still, bias due to subjective reporting of work exposures may exist. However, the questionnaire (QPS_Nordic_) has been designed with the intention to attenuate reporting bias due to mood state and personality dispositions [14]. Participants are asked to rate items by frequency of occurrence rather than the extent to which they agree/disagree with the item content, and items are carefully worded to describe situations without referring to negative or positive emotions (satisfaction/dissatisfaction) resulting from these situations.

The present study was based on data of exposure levels measured once. During the follow-up period of (maximum) 10 years, some subjects may have experienced - for the most of this period - a different exposure level than the level reported at baseline. Thus, misclassification according to exposure level may have occurred. To address this issue, future studies with designs incorporating measures of psychological/social and mechanical exposures at several time points seem warranted. Additionally, by accounting for exposure level at multiple time points, subsequent studies should be able to address “dose-response” relationships more appropriately. Risk of disability retirement may be different for subjects reporting, for instance, constant high level of role conflict compared to subjects reporting alternations between high and middle levels of this exposure, across measurement points.

Future studies may also want to stratify by occupational group/branch to investigate if the identified predictors contribute differently to disability retirement in different occupations. In the present study, some of the branches were represented by relatively few companies with few disability cases. Thus, statistical power issues did not allow for detailed analyses, i.e. testing the effects of each exposure on disability retirement while adjusting for relevant covariates in *each* branch.

## Conclusions

The present study contributes to existing work disability-research by elucidating contributions of factors which previously have been largely unexplored or incorporated in broader assessments of psychological and social exposures. Several specific predictors were revealed, and knowledge of these may represent an advantage to organizational improvement programs aiming to prevent premature exit from the work force. The results of the present study suggest that practical interventions should particularly focus on reducing occupational role conflicts and physical workload, and on promoting fair leadership, positive challenge at work, and control over work intensity. It remains an important issue however if *modification* of the identified contributors may result in lowered risk of disability retirement.

## Abbreviations

CI: Confidence interval
HR: Hazard ratio
N: Number
OR: Odds ratio
SD: Standard deviation

## References

[1] World Health Organization (2001). International Classification of Functioning, Disability and Health - ICF.

[2] Dragano N, Schneider L. Psychosoziale Arbeitsbelastungen als Prädiktoren der krankheitsbedingten Frühberentung. [Work related psychosocial factors and the risk of early disability pensioning: a contribution to assessing the need for rehabilitation]. Rehabilitation. 2011; doi:10.1055/s-0030-1270431. 10.1055/s-0030-1270431 21321822

[3] Canivet C, Choi B, Karasek R, Moghaddassi M, Staland-Nyman C, Ostergren PO. Can high psychological job demands, low decision latitude, and high job strain predict disability pensions? A 12-year follow-up of middle-aged Swedish workers. Int Arch Occup Environ Health. 2013; doi:10.1007/s00420-012-0766-4. 10.1007/s00420-012-0766-4 22476722

[4] Labriola M, Lund T. Self-reported sickness absence as a risk marker of future disability pension. Prospective findings from the DWECS/DREAM study 1990–2004. Int J Med Sci. 2007; doi:10.7150/ijms.4.153. 10.7150/ijms.4.153 PMC1885553 17554400

[5] Lahelma E, Laaksonen M, Lallukka T, Martikainen P, Pietiläinen O, Saastamoinen P et al. Working conditions as risk factors for disability retirement: a longitudinal register linkage study. BMC Public Health. 2012; doi:10.1186/1471-2458-12-309. 10.1186/1471-2458-12-309 PMC3438015 22537302

[6] Karasek R, Brisson C, Kawakami N, Houtman I, Bongers P, Amick B. The Job Content Questionnaire (JCQ): an instrument for internationally comparative assessments of psychosocial job characteristics. J Occup Health Psychol. 1998; doi:10.1037/1076-8998.3.4.322. 10.1037//1076-8998.3.4.322 9805280

[7] Ylipaavalniemi J, Kivimaki M, Elovainio M, Virtanen M, Keltikangas-Jarvinen L, Vahtera J. Psychosocial work characteristics and incidence of newly diagnosed depression: a prospective cohort study of three different models. Soc Sci Med. 2005; doi:10.1016/j.socscimed.2004.11.038. 10.1016/j.socscimed.2004.11.038 15847966

[8] Christensen JO, Knardahl S. Work and neck pain: A prospective study of psychological, social, and mechanical risk factors. PAIN. 2010; doi:10.1016/j.pain.2010.07.001. 10.1016/j.pain.2010.07.001 20655144

[9] Emberland JS, Knardahl S. Contribution of psychological, social, and mechanical work exposures to low work ability: a prospective study. J Occup Environ Med. 2015; doi:300–314.10.1097/jom.0000000000000353. 10.1097/JOM.0000000000000353 PMC4351996 25470453

[10] Hagen KB, Tambs K, Bjerkedal T. A prospective cohort study of risk factors for disability retirement because of back pain in the general working population. Spine. 2002; doi:10.1097/00007632-200208150-00019. 10.1097/00007632-200208150-00019 12195073

[11] Krokstad S, Johnsen R, Westin S. Social determinants of disability pension: a 10-year follow-up of 62000 people in a Norwegian county population. Scand J Public Health. 2002; doi: 10.1093/ije/31.6.1183. 10.1093/ije/31.6.1183 12540720

[12] Nielsen MB, Christiansen S, Indregard A-MR, Emberland JS, Elka S, Knardahl S. The new workplace II: protocol for a prospective full-panel registry study of work factors, sickness absence, and exit from working life among Norwegian employees. SpringerPlus. 2016; doi:10.1186/s40064-016-1896-z. 10.1186/s40064-016-1896-z PMC4771681 27026937

[13] Norwegian Labour and Welfare Administration: Benefits and services. https://www.nav.no/en/Home/Benefits+and+services/Pensions+and+pension+application+from+outside+Norway/Disability+benefit#chapter-1 (2011). Accessed 1 Feb 2016.

[14] Dallner M, Elo A-L, Gamberale F, Knardahl S, Hottinen V, Lindström K (2000). Validation of the General Nordic Questionnaire (QPS_Nordic_) for psychological and social factors at work.

[15] Wännström I, Peterson U, Asberg M, Nygren A, Gustavsson JP. Psychometric properties of scales in the General Nordic Questionnaire for Psychological and Social Factors at Work (QPS_Nordic_): confirmatory factor analysis and prediction of certified long-term sickness absence. Scand J Psychol. 2008; doi:10.1111/j.1467-9450.2008.00697.x. 10.1111/j.1467-9450.2008.00697.x 19037910

[16] Therneau T, Lumley T. Survival: Survival Analysis. 2016. https://cran.r-project.org/web/packages/survival/index.html. Accessed 5 June 2016.

[17] Korn EL, Graubard BI, Midthune D (1997). Time-to-event analysis of longitudinal follow-up of a survey: choice of the time-scale. Am J Epidemiol.

[18] Kivimäki M, Nyberg ST, Batty GD, Fransson EI, Heikkilä K, Alfredsson L et al. Job strain as a risk factor for coronary heart disease: a collaborative meta-analysis of individual participant data. Lancet. 2012; doi:10.1016/S0140-6736(12)60994-5.

[19] Virtanen M, Ferrie JE, Singh-Manoux A, Shipley MJ, Vahtera J, Marmot MG et al. Overtime work and incident coronary heart disease: the Whitehall II prospective cohort study. Eur Heart J. 2010; doi:10.1093/eurheartj/ehq124. 10.1093/eurheartj/ehq124 PMC2903713 20460389

[20] Allebeck P, Mastekaasa A. Chapter 5. Risk factors for sick leave - general studies. Scand J Public Health. 2004; doi:10.1080/14034950410021853. 10.1080/14034950410021853 15513654

[21] Goldberg P, Guéguen A, Schmaus A, Nakache J-P, Goldberg M. Longitudinal study of associations between perceived health status and self reported diseases in the French Gazel cohort. J Epidemiol Community Health. 2001; doi:10.1136/jech.55.4.233. 10.1136/jech.55.4.233 PMC1731872 11238577

[22] Westerlund H, Kivimaki M, Singh-Manoux A, Melchior M, Ferrie JE, Pentti J et al. Self-rated health before and after retirement in France (GAZEL): a cohort study. Lancet. 2009; doi:10.1016/s0140-6736(09)61570-1. 10.1016/S0140-6736(09)61570-1 19897238

[23] Rothman KJ, Greenland S, Lash TL (2008). Modern Epidemiology.

[24] Karasek RA. Job Demands, Job Decision Latitude, and Mental Strain: Implications for Job Redesign. Admin Sci Q. 1979; doi:10.2307/2392498.

[25] Laine S, Gimeno D, Virtanen M, Oksanen T, Vahtera J, Elovainio M et al. Job strain as a predictor of disability pension: the Finnish Public Sector Study. J Epidemiol Community Health. 2009; doi:10.1136/jech.2007.071407. 10.1136/jech.2007.071407 18768568

[26] Mäntyniemi A, Oksanen T, Salo P, Virtanen M, Sjösten N, Pentti J et al. Job strain and the risk of disability pension due to musculoskeletal disorders, depression or coronary heart disease: a prospective cohort study of 69 842 employees. Occup Environ Med. 2012; doi:10.1136/oemed-2011-100411. 10.1136/oemed-2011-100411 22573793

[27] Lund T, Iversen L, Poulsen KB. Work environment factors, health, lifestyle and marital status as predictors of job change and early retirement in physically heavy occupations. Am J Ind Med. 2001; doi:10.1002/ajim.1084. 10.1002/ajim.1084 11494344

[28] Tengland PA. The concept of work ability. J Occup Rehabil. 2011; doi:10.1007/s10926-010-9269-x. 10.1007/s10926-010-9269-x 21052807

[29] Sterud T. Work-related psychosocial and mechanical risk factors for work disability: a 3-year follow-up study of the general working population in Norway. Scand J Work Environ Health. 2013; doi:10.5271/sjweh.3359. 10.5271/sjweh.3359 23529701

[30] Hinkka K, Kuoppala J, Vaananen-Tomppo I, Lamminpaa A. Psychosocial work factors and sick leave, occupational accident, and disability pension: a cohort study of civil servants. J Occup Environ Med. 2013; doi:10.1097/JOM.0b013e31827943fe. 10.1097/JOM.0b013e31827943fe 23364212

[31] Sinokki M, Hinkka K, Ahola K, Gould R, Puukka P, Lonnqvist J et al. Social support as a predictor of disability pension: the Finnish Health 2000 study. J Occup Environ Med. 2010; doi:10.1097/JOM.0b013e3181e79525. 10.1097/JOM.0b013e3181e79525 20595913

[32] Mein G, Martikainen P, Stansfeld SA, Brunner EJ, Fuhrer R, Marmot MG. Predictors of early retirement in British civil servants. Age Ageing. 2000; doi:10.1093/ageing/29.6.529. 10.1093/ageing/29.6.529 11191246

[33] Campos-Serna J, Ronda-Pérez E, Artazcoz L, Moen BE, Benavides FG. Gender inequalities in occupational health related to the unequal distribution of working and employment conditions: a systematic review. Int J Equity Health. 2013; doi:10.1186/1475-9276-12-57. 10.1186/1475-9276-12-57 PMC3765149 23915121

[34] Criqui MH (1979). Response bias and risk ratios in epidemiologic studies. Am J Epidemiol.

[35] Laaksonen M, Aittomäki A, Lallukka T, Rahkonen O, Saastamoinen P, Silventoinen K et al. Register-based study among employees showed small nonparticipation bias in health surveys and check-ups. J Clin Epidemiol. 2008; doi:10.1016/j.jclinepi.2007.09.010. 10.1016/j.jclinepi.2007.09.010 18486445

[36] Nielsen MB, Knardahl S. The healthy worker effect: do health problems predict participation rates in, and the results of, a follow-up survey? Int Arch Occup Environ Health. 2015; doi:10.1007/s00420-015-1066-6. 10.1007/s00420-015-1066-6 26105125

[37] Pearce N, Checkoway H, Kriebel D. Bias in occupational epidemiology studies. Occup Environ Med. 2007; doi:10.1136/oem.2006.026690. 10.1136/oem.2006.026690 PMC2078501 17053019

[38] Podsakoff PM, MacKenzie SB, Lee JY, Podsakoff NP. Common method biases in behavioral research: a critical review of the literature and recommended remedies. J Appl Psychol. 2003; doi:10.1037/0021-9010.88.5.879. 10.1037/0021-9010.88.5.879 14516251

